# Ablation of the Right Cardiac Vagus Nerve Reduces Acetylcholine Content without Changing the Inflammatory Response during Endotoxemia

**DOI:** 10.3390/ijms19020442

**Published:** 2018-02-01

**Authors:** Konstanze Plaschke, Thuc Quyen Monica Do, Florian Uhle, Thorsten Brenner, Markus A. Weigand, Jürgen Kopitz

**Affiliations:** 1 Department of Anesthesiology, Heidelberg University Hospital, Im Neuenheimer Feld 110, D-69120 Heidelberg, Germany; mdo173@hotmail.com (T.Q.M.D.); florian.uhle@med.uni-heidelberg.de (F.U.); Thorsten.brenner@med.uni-heidelberg.de (T.B.); Markus.weigand@med.uni-heidelberg.de) (M.A.W.); 2 Department of Pathology, Heidelberg University Hospital, Im Neuenheimer Feld 224, D-69120 Heidelberg, Germany; Juergen.kopitz@med.uni-heidelberg.de

**Keywords:** sepsis, endotoxemia, right cardiac vagus nerve resection, inflammation, hemodynamic parameters

## Abstract

Acetylcholine is the main transmitter of the parasympathetic vagus nerve. According to the cholinergic anti-inflammatory pathway (CAP) concept, acetylcholine has been shown to be important for signal transmission within the immune system and also for a variety of other functions throughout the organism. The spleen is thought to play an important role in regulating the CAP. In contrast, the existence of a “non-neuronal cardiac cholinergic system” that influences cardiac innervation during inflammation has been hypothesized, with recent publications introducing the heart instead of the spleen as a possible interface between the immune and nervous systems. To prove this hypothesis, we investigated whether selectively disrupting vagal stimulation of the right ventricle plays an important role in rat CAP regulation during endotoxemia. We performed a selective resection of the right cardiac branch of the Nervus vagus (VGX) with a corresponding sham resection in vehicle-injected and endotoxemic rats. Rats were injected with lipopolysaccharide (LPS, 1 mg/kg body weight, intravenously) and observed for 4 h. Intraoperative blood gas analysis was performed, and hemodynamic parameters were assessed using a left ventricular pressure-volume catheter. Rat hearts and blood were collected, and the expression and concentration of proinflammatory cytokines using quantitative reverse transcription polymerase chain reaction and enzyme-linked immunosorbent assay were measured, respectively. Four hours after injection, LPS induced a marked deterioration in rat blood gas parameters such as pH value, potassium, base excess, glucose, and lactate. The mean arterial blood pressure and the end-diastolic volume had decreased significantly. Further, significant increases in blood cholinesterases and in proinflammatory (IL-1β, IL-6, TNF-α) cytokine concentration and gene expression were obtained. Right cardiac vagus nerve resection (VGX) led to a marked decrease in heart acetylcholine concentration and an increase in cardiac acetylcholinesterase activity. Without LPS, VGX changed rat hemodynamic parameters, including heart frequency, cardiac output, and end-diastolic volume. In contrast, VGX during endotoxemia did not significantly change the concentration and expression of proinflammatory cytokines in the heart. In conclusion we demonstrate that right cardiac vagal innervation regulates cardiac acetylcholine content but neither improves nor worsens systemic inflammation.

## 1. Introduction

Despite intensive research, sepsis and septic shock still represent the major causes of death in intensive care units (ICU) [1,2,3]. In the ICU, sepsis-induced cardiac dysfunction develops frequently [4,5,6]. One important aspect of septic cardiomyopathy is autonomic dysfunction, which develops when the nerves of the autonomic nervous system (sympathetic and/or parasympathetic) are damaged. For instance, autonomic dysfunction in septic cardiomyopathy [7] is reflected by decreased heart rate variability (which is defined as the variation in the time interval between heartbeats).

Otherwise, as is well known, sepsis is mainly characterized by the occurrence of strong inflammatory processes [8].

The cholinergic anti-inflammatory pathway (CAP) concept can be defined as the missing link between regulation of the inflammatory response and the neural circuit. According to the CAP concept, acetylcholine—the principal vagal neurotransmitter—was able to reduce in vitro proinflammatory cytokine levels in response to endotoxin in human macrophages and in vivo through direct electrical stimulation of the efferent vagal nerve in rats [9]. These experiments highlighted the link between the immune and vegetative nervous systems, and the concept of the cholinergic anti-inflammatory reflex was postulated [10]. Accordingly, it was further postulated that the spleen is required for the cytokine regulatory effect [11]. However, more recent studies have provided evidence that the vagal preganglionic terminals do not synapse with the sympathetic neurons innervating the spleen [12,13]. In this context, an alternative idea [14] was hypothesized, introducing the heart instead of the spleen as the possible interface between the immune and nervous systems. Therefore, in the present paper we sought to determine whether the heart’s parasympathetic innervation plays an important role in regulating (systemic) inflammation during endotoxemia.

## 2. Results

### 2.1. Intraoperative Blood Gas Analysis 

Before administering LPS (T1, baseline), no significant differences between the groups were determined for the following parameters: pH value, pCO_2_, pO_2_, bicarbonate (HCO_3_^−^), base excess (BE), hematocrit (Hk), hemoglobin (Hb), potassium (K^+^), glucose (Glu), and lactate (Lac) concentrations. Thus, all these measured parameters were in the physiological range.

At the end of the experiments (T5), however, marked, significant changes were found in pH value, K^+^, BE, bicarbonate, glucose, and lactate concentration in endotoxemic rats compared to saline-injected rats (^§^: *p* < 0.05, Table 1) between experimental groups III and I and IV and II, respectively.

Resection of the right cardiac vagus nerve (VGX), however, did not show significant changes in blood gas parameters (Table 1) under saline or under LPS conditions.

### 2.2. Acetylcholine and Cholinesterase (CHE) Activity 

**Figure 1:**
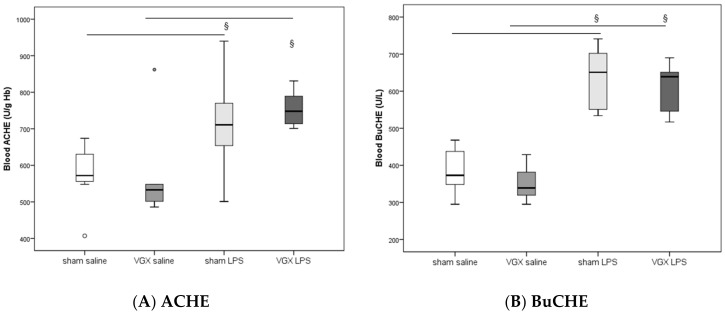
Blood cholinesterase (CHE) activities at the end of the experiments. Blood enzyme activity presented as median (IQR = Inter Quartile Range) at 4 h after LPS injection (T5). No significant effect of right cardiac vagus nerve resection (VGX) was observed (*p* > 0.05). ^§^: significant LPS effect for (**A**) acetylcholinesterase (ACHE, U/g Hb) activity (sham saline versus sham LPS, *p* = 0.031, and VGX saline versus VGX LPS, *p* < 0.001) and for (**B**) butyrylcholinesterase (BuCHE, U/L) activity (sham saline versus sham LPS, *p* < 0.001, and VGX saline versus VGX LPS, *p* < 0.001). The circles present outliers.

In rat blood, acetylcholinesterase (ACHE) and butyrylcholinesterase (BuCHE) activities were measured four hours after injecting LPS (T5). The activity of both enzymes was significantly higher after LPS stimulation, irrespective of the selective vagus intervention, than in the corresponding saline groups (^§^: *p* ≤ 0.05, Figure 1). Vagotomy of the right cardiac vagus nerve alone did not induce any change in CHE activity (*p* > 0.05, Figure 1).

**Figure 2:**
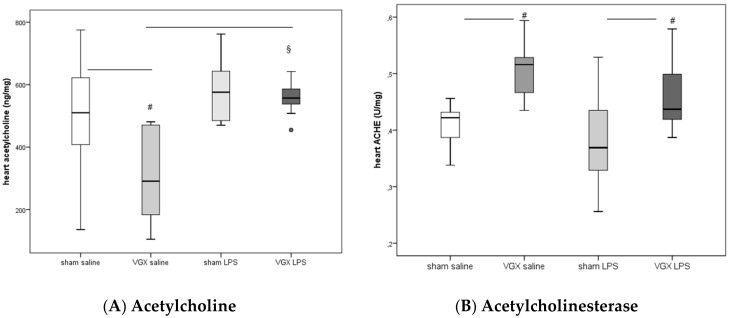
(**A**) Acetylcholine (ACH) and (**B**) acetylcholinesterase (ACHE) in rat heart at the end of the experiments. Data presented as median (IQR = Inter Quartile Range) at 4 h after LPS injection (T5). ^#^: significant effect of right cardiac vagus nerve resection (ACH in ng/mg protein: *p* = 0.038, ACHE in U/mg protein: *p* = 0.004 (sham saline versus VGX saline) and *p* = 0.05 (sham LPS versus VGX LPS). ^§^: significant LPS effect: (VGX saline versus VGX LPS, *p* = 0.016). The circles present outliers.

LPS significantly increased acetylcholine in rat myocardium after a selective vagotomy of the right branch of the cardiac vagus nerve (VGX, Figure 2A: ^§^: *p* < 0.05).

A right cardiac vagus nerve resection (VGX) without LPS significantly reduced the ACH concentration in myocardial tissue, (^#^: *p* ≤ 0.05, Figure 2A). When administered together with LPS, however, this effect was diminished (*p* > 0.05, Figure 2A).

ACHE activity in heart tissue increased after selective VGX (^#^: *p* ≤ 0.05, Figure 2B) independently of whether rats were injected with saline or LPS. LPS had no significant effect on myocardial ACHE activity (*p* > 0.05).

### 2.3. Plasma Corticosterone Concentration

Plasma corticosterone concentration (mean ± standard error of means, ng/mL) was not significantly different between the four groups (I–IV) at the end of the experimental period: (I) 278.4 ± 10.9, (II) 283.0 ± 9.7, (III) 326.4 ± 14.2, and (IV) 303.8 ± 14.9 ng/mL.

### 2.4. Cytokine Concentration and Expression

**Figure 3:**
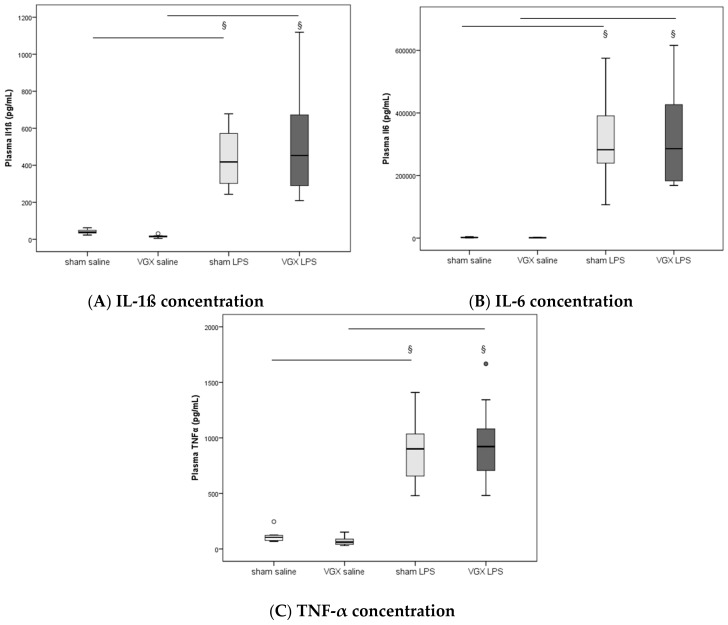
Plasma pro-inflammatory cytokines ((**A**) IL-1ß, (**B**) IL-6, (**C**) TNF-α) at the end of the experiment. Plasma data presented as median (IQR = Inter Quartile Range, in pg/mL) at 4 h after LPS injection (T5). No significant effect of nerve resection (VGX) was obtained (*p* > 0.05). Significant LPS effect (^§^: sham LPS versus sham saline and VGX LPS versus VGX saline, *p* ≤ 0.001 for both) was detected in all plasma proinflammatory cytokines measured. The circles present outliers.

**Figure 4:**
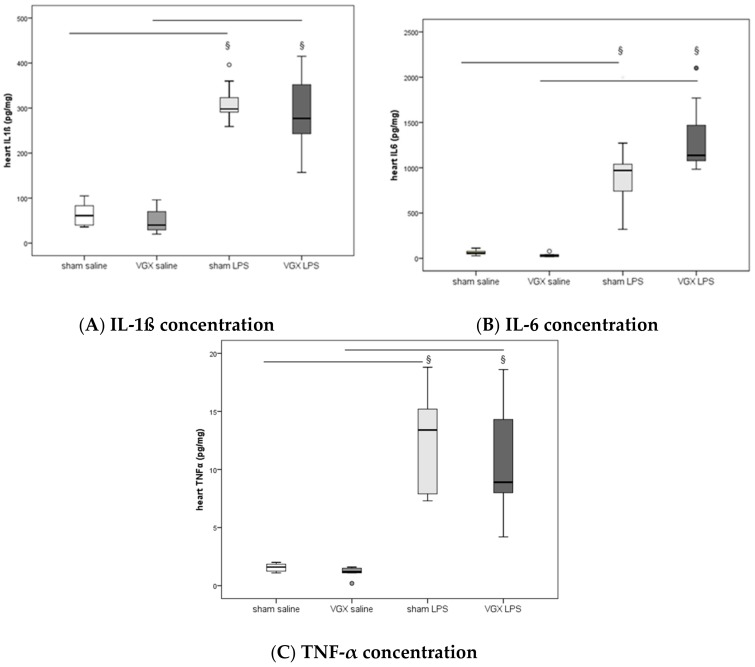
Heart proinflammatory cytokine concentrations ((**A**) IL-1ß, (**B**) IL-6, (**C**) TNF-α) at the end of the experiments. Heart data presented as median (IQR = Inter Quartile Range, in pg/mg protein) at 4 h after LPS injection (T5). No significant effect of nerve resection was obtained (*p* > 0.05). Significant LPS effect (^§^: sham LPS versus sham saline and VGX LPS versus VGX saline, *p* ≤ 0.001 for both) was detected in all tissue proinflammatory cytokines measured. The circles present outliers.

**Figure 5:**
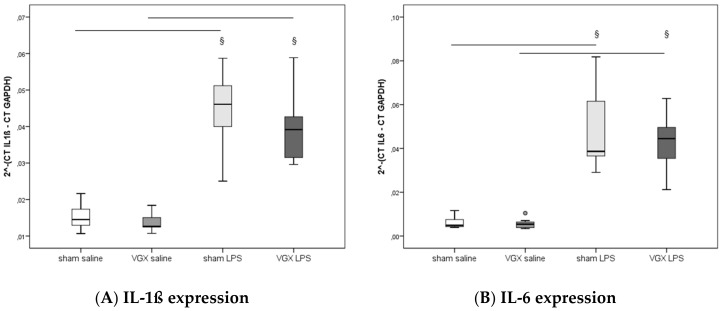

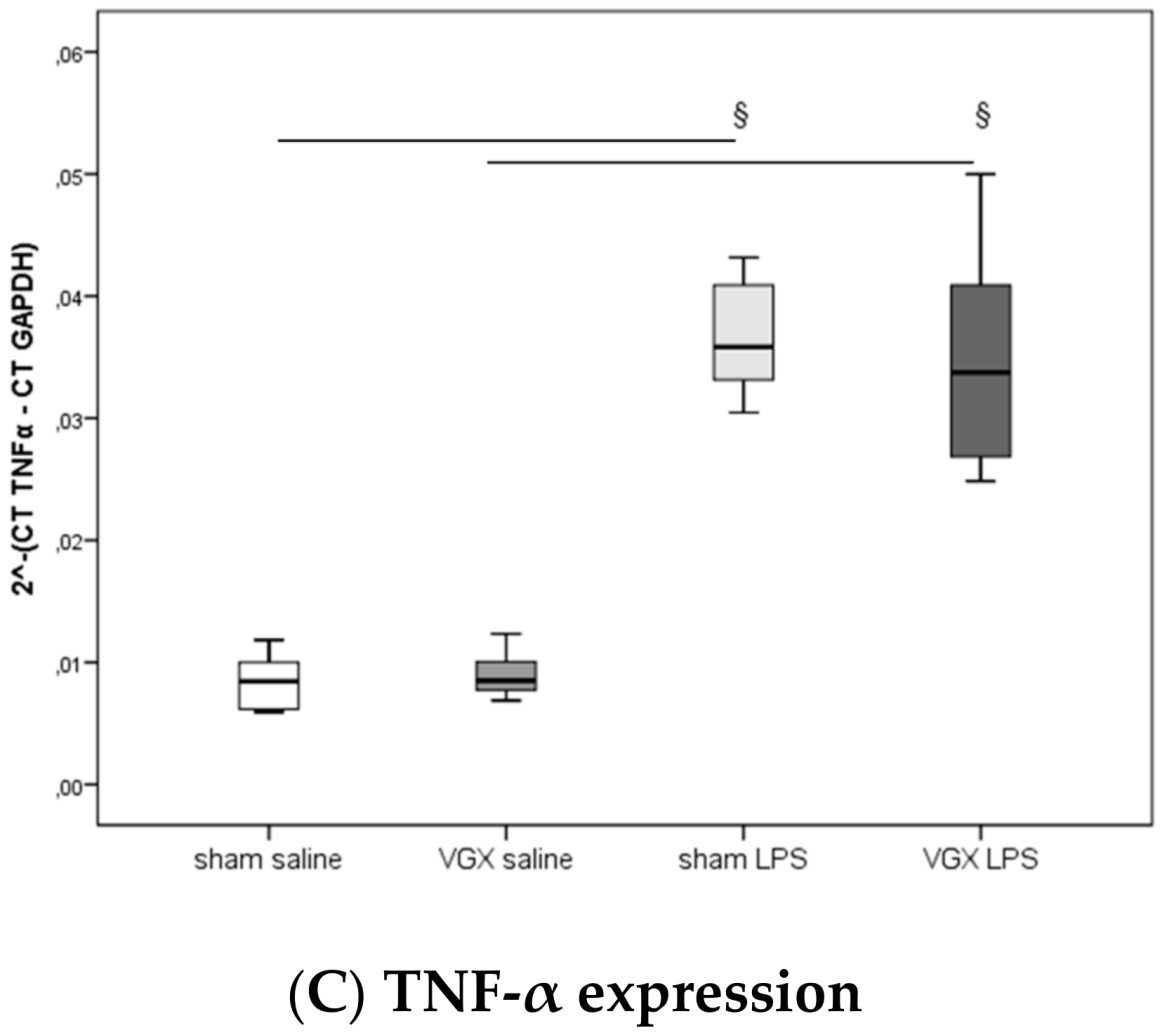
Cardiac proinflammatory mRNA expression ((**A**) IL-1ß, (**B**) IL-6, (**C**) TNF-α) at the end of the experiments. Data presented as normalized expression (median, IQR = Inter Quartile Range) at 4 h after LPS injection (T5). No significant effect of right cardiac vagus nerve resection was observed (*p* > 0.05). Significant LPS effect (^§^: sham LPS versus sham saline and VGX LPS versus VGX saline, *p* ≤ 0.001) was detected in all tissue proinflammatory cytokine mRNA levels measured. The circles present outliers.

We measured the proinflammatory cytokines IL-1β, IL-6, and TNF-α in rat plasma and myocardial tissue. All three proinflammatory cytokines had significantly increased after LPS treatment (^§^, *p* < 0.05), both in gene expression in the heart and protein levels in plasma (Figure 3A–C) and myocardium (Figure 4A–C and Figure 5A–C). A selective resection of the right cardiac vagus nerve (VGX) did not change the inflammatory response (*p* > 0.05).

### 2.5. Cardiovascular and Hemodynamic Parameters

At baseline (Table 2, column T1), no significant differences in the cardiovascular and hemodynamic parameters were observed between animal groups (*p* > *0.05)*.

After administering LPS, and irrespective of a vagal intervention, animals exhibited a significant decrease in mean arterial blood pressure (MAP, ^§^: *p*_1_ < 0.05, Table 2, minus 35% in sham-operated rats and −42% in VGX rats) at T5 (treatment effect). Rat heart frequency (HF) and cardiac output (CO) had not changed significantly after LPS administration compared to animals with saline injection. The end-diastolic volume (EDV) was significantly decreased minus 40% in VGX LPS rats (Table 2, ^§^: *p*_1_ < 0.05).

After administering LPS and irrespective of vagal intervention, animals exhibited a significant decrease in mean arterial blood pressure (MAP, ^§^: *p* < 0.05). The end-diastolic volume (EDV) was significantly decreased in VGX LPS rats compared to VGX saline ^§^: *p* < 0.05). In saline-injected rats, the effect of VGX was obtained in heart frequency (HF), cardiac output (CO), and EDV (^#^: *p* < 0.05). Under LPS, the effect of VGX was only significantly pronounced in rat HF (comparison group IV versus III, ^##^: *p* < 0.05).

A right cardiac vagus nerve resection (VGX) significantly (^#^: *p*_2_ < 0.05) reduced (i) HF under saline and LPS conditions, and (ii) CO under saline injection in rats. EDV, however, was significantly increased in VGX rats under the condition of saline injection (Table 2, ^#^: *p*_2_ = 0.05).

**Figure 6:**
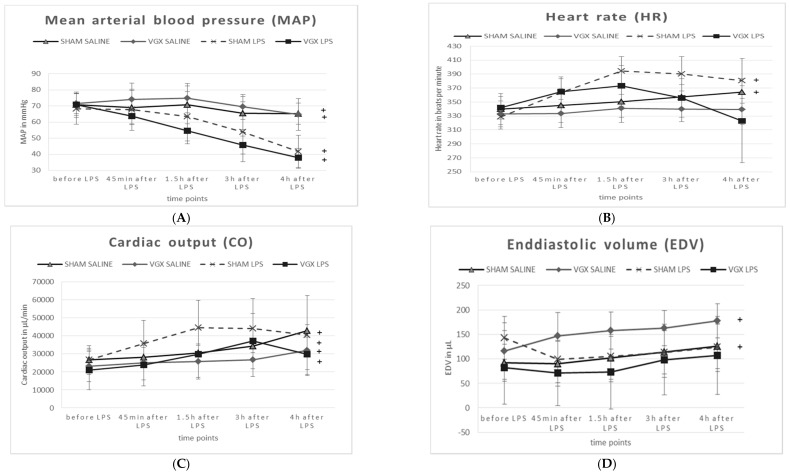
Effect over the experimental time in cardiovascular and hemodynamic measurements ((**A**) mean arterial blood pressure, (**B**) heart rate, (**C**) cardiac output, (**D**) enddiastolic volume, mean ± SD) in control and endotoxemic rats and after right cardiac vagus nerve resection (VGX). Data are represented as means ± SD. Changes during the experimental period are presented: (end of the experiment versus data before LPS): “+”: time effect *p* ≤ 0.05.

Compared to baseline (T5 versus T1, time effect), the MAP was significantly decreased and the CO was significantly increased in all animal groups (Figure 6A,C). HF was only significantly increased (*p* < 0.05) in sham-operated rats, whereas VGX seemed to attenuate this effect (Figure 6B). A significant increase in end-diastolic volume (EDV) over time was observed only in saline-, but not in LPS-injected rats (Figure 6D).

## 3. Discussion

The main result of the present study showed that vagal outflow is related to cardiac acetylcholine content, but neither improves nor worsens systemic inflammation.

### 3.1. LPS Model

Apart from the LPS models, the other main animal model of sepsis is the surgical model (e.g., the cecal ligation and puncture (CLP) model), which is also clinically relevant as this model induces a polymicrobial sepsis that mimics human sepsis [15,16]. However, (i) owing to the high acceptance of LPS murine models (for review see: [16]) analogous to human sepsis, and (ii) to compare our present results with the results of our preliminary study in which LPS was also used at a concentration of 1 mg/kg body weight [17], we decided to employ the rat LPS model to mimic acute cardiovascular, hemodynamic, and inflammatory changes. In previous experiments [17] we have already performed survival analyses up to 6 hours after LPS (1 mg/kg body weight, intravenously, i.v.). This previously performed study indicated that after intravenous application of 1 mg/kg LPS, rats are not able to survive longer than 5–6 h. Consequently, we selected an observation time of 4 h after LPS to ensure survival of all animals under the selected experimental and anesthetic conditions. Because we did not performed survival analysis in the present study, no conclusions could be drawn concerning mortality after administering lipopolysaccharide (LPS) in this actually presented manuscript.

In accordance with the literature [17,18], endotoxemia in the present model was characterized by cardiovascular and hemodynamic impairment. In addition, we demonstrated that injecting LPS in rodents produces a pronounced systemic inflammatory reaction in the form of markedly increased expression and concentration of proinflammatory cytokines, as also shown previously by other studies [19,20,21,22].

Summarizing the results after LPS injection, we demonstrated that a single LPS dose (1 mg/kg body weight administered intravenously) was able to mimic some of the most important signs that are observed in septic patients, and therefore it is an appropriate animal model to use for studying open questions in inflammation-induced, cardiovascular alterations. Nevertheless, with only 4 h of observation time, we can only assess the very early changes using our acute LPS model. To assess compensatory mechanisms occurring delayed, further models must be utilized.

### 3.2. Effect of Selective Right Cardiac Vagotomy (VGX)

#### 3.2.1. Model of Right Cardiac Nerve Resection (VGX)

Early studies considered that fine, silver-stained fibers coursing among ventricular myocardial cells were most likely cardiac parasympathetic postganglionic fibers [23]. To our knowledge, we have performed a selective right cardiac vagotomy in a rat for the first time. Therefore, the question arose as to whether this procedure was performed in a surgically correct way. Although we performed Bielschowsky silver staining [24] for nerve fibers, it is not possible to discriminate histopathologically the right vagus nerve from the right cardiac branch of the vagus nerve. However, the present results showing a reduction in ACH and an increase in ACHE in rat heart after VGX indicated indirectly that we cut the right cardiac branch of the Nervus vagus correctly.

It is known that inflammation can be influenced by stress response through surgical intervention. Therefore, we measured the plasma corticosterone concentration. No changes in corticosterone plasma levels were observed between the groups; thus, possible differences between sham-operated and the right cardiac vagus nerve resected rats were not directly associated with surgical stress-related deterioration in the hypothalamic-pituitary-adrenal (HPA) axis and related peripheral corticosterone concentration.

#### 3.2.2. Selective Right Cardiac Vagus Nerve Resection (VGX) Reduced Cardiac Function

Selective VGX depressed cardiac function at the end of the experimental period: the VGX effect was mainly pronounced for HF, CO, and EDV in the present study. Alternative pathways partially compensating for the detrimental impact of a systemic loss of vagal tone were described in the studies of Horn and Friedman and Martelli et al. [25,26]. For technical and preparatory reasons, it is not possible to cut both the right and the left cardiac branches in rats in one experimental surgical procedure in order to completely exclude heart parasympathetic innervation. We showed here that the right vagus nerve does not appear to be involved in anti-inflammatory effects. However, the right cardiac vagus nerve is the main factor driving the parasympathetic component of heart rate variability (HRV) [27]. Some researchers propose that parasympathetic HRV activity reflects cholinergic anti-inflammatory pathway activity [28]. However, our present data suggest that the parasympathetic system is much more compartmentalized, meaning that vagal outflow to the heart (measured by HRV) does not reflect vagal outflow to other (inflammatory) organs.

Under LPS-induced inflammation, the MAP was reduced, irrespective of whether the vagus nerve was intact or resected (at the end of the experiments). The persistent drop in HF after VGX appears somehow paradoxical, but might be based on an (over)compensatory loss of sympathetic tone. As a consequence, CO is also reduced, which might reduce the supply of substrates to the tissue, as indicated by unchanged pO_2_ and significantly lower glucose levels in animals after LPS and VGX. Thus, because organ perfusion is compromised as a result of endotoxemia, increased lactate levels were observed. However, whether the observed influence of the vagus nerve is due to changes in cardiac metabolism or a result of hemodynamic alterations should be the subject of further research.

### 3.3. Right Cardiac Vagus Nerve Resection (VGX) Did Not Influence Inflammatory Parameters

It was shown in previous experiments that myocardial cells are capable of secreting pro-inflammatory cytokines upon sensing of circulating inflammatory mediators or pathogen-associated molecular pattern, e.g., LPS [29].

Contrary to our primary hypothesis about a significant role of the heart in CAP, our present results showed an unchanged inflammatory response after VGX, as assessed by proinflammatory cytokine levels of IL-1β, IL-6, and TNF-α in the plasma and ventricular tissues. Analyses of myocardial expression of these genes are in concordance with the protein levels.

In contrast to nerve resection, vagus nerve stimulation represents a common therapy option under some clinical circumstances, for instance, epilepsy and depression [30]. Electrical stimulation of the efferent vagal nerve fibers with low frequency is thought to possess anti-inflammatory properties and is able to activate the “cholinergic anti-inflammatory reflex” [31]. In previous studies—likewise using a “vagus-stimulation” animal model—Schulte et al. [17] showed an overall beneficial effect of vagal stimulation on hemodynamic parameters. Furthermore, they reported reduced proinflammatory cytokine levels of IL-1β and TNF-α in plasma and cardiac tissue on mRNA and protein levels. To reverse both the hemodynamic and immunologic effects of diminished vagal tone, even short-term stimulation of the Nervus vagus seems to be sufficient during the LPS infusion. During sepsis and septic shock [17,32], an evidence for a potential use of vagus nerve stimulators was provided to reestablish the lacking parasympathetic innervation in critically ill patients. We did not perform additional nerve stimulation experiments on selective right cardiac vagus branches because we did not find any changes in hemodynamic and inflammatory parameters after VGX in our study. In contrast to “vagus stimulation” experiments, the focus of the present investigation addressed the question of whether a selective resection of the cardiac branch of the right Nervus vagus plays a significant role in the CAP because the involvement of the spleen in the CAP has been discussed controversially, focusing on the anatomical absence of parasympathetic innervation of the organ [12,13,33,34].

Summarizing the main results: our present study demonstrates significant changes in heart ACH and ACHE after ablation of the right cardiac vagus nerve. However, we did not find evidence for a significant role of the heart in the cholinergic anti-inflammatory pathway.

## 4. Materials and Methods 

### 4.1. Animals

The experimental protocol was approved by the review committee of the Medical Faculty of the University of Heidelberg (Germany) and complied with the guidelines of the responsible national government agency (Regierungspraesidium, Karlsruhe, Germany, 21/10/2015, G-169/15) and with international standards.

The studies were performed on adult male Wistar rats (Janvier Labs from Saint-Berthevin Cedex, France), weighing 350–390 g. They were housed in individual cages in a temperature-controlled room at 22 ± 0.5 °C with a reversed day–night cycle (12 h:12 h, light on at 7 p.m.). Free access to food (LASQc diet, LASvendi‚ Soest, Germany) and water was allowed throughout the experimental period.

A total of 32 adult male rats were randomly allocated to 4 groups:

(I) Sham nerve resection group with 1 mL i.v. saline (*n =* 7) = (sham saline)
(II) Nerve resection group: with 1 mL saline i.v. (*n =* 7) = (VGX saline)
(III) Sham resection group + LPS (1 mg/kg body weight i.v.) (*n =* 9) (sham LPS)
(IV) Nerve resection group + LPS (1 mg/kg body weight i.v.) (*n =* 9) (VGX LPS)

(Abbrev.: LPS: lipopolysaccharide, i.v.: intravenously, VGX: resection of the right cardiac vagus nerve)

### 4.2. Surgical Procedures

After induction of anesthesia with 4 vol. % sevoflurane (Abbott, Wiesbaden, Germany) via a vaporizer (Dräger, Lübeck, Germany) and oxygen/nitrous oxide (O_2_:N_2_O = 30:70), anesthesia was maintained over the entire experimental period with 3.5 vol. % sevoflurane and oxygen/nitrous oxide (O_2_:N_2_O = 30:70).

After endotracheal intubation with a 16-G catheter, rats were ventilated with 60/min breathing frequency using a rodent respirator (Föhr Medical Instruments, Seeheim-Jugenheim, Germany). After subcutaneous electrode placement, the electrocardiogram was recorded. Tympanal temperature was monitored and maintained at around 37 °C by using a heating pad and a heater.

In all animals, catheters were placed in the A. and V. femoralis sinistra (5.0 nylon thread) for administering drugs, for drawing blood samples, or for blood gas analysis. We have a very long experience in preparing femoral vein instead of tail vein. Under the conditions of prolonged anesthesia, the femoral vein is easy and reproducible to prepare, especially if an agent/medication etc. had to be infused.

Thereafter, the A. carotis communis dextra was prepared and a microtip pressure-volume catheter (SPR-838, Millar Instruments, Houston, TX, USA) was inserted through the A. communis dextra and advanced into the left ventricle for continuous hemodynamic measurement.

**Figure 7:**
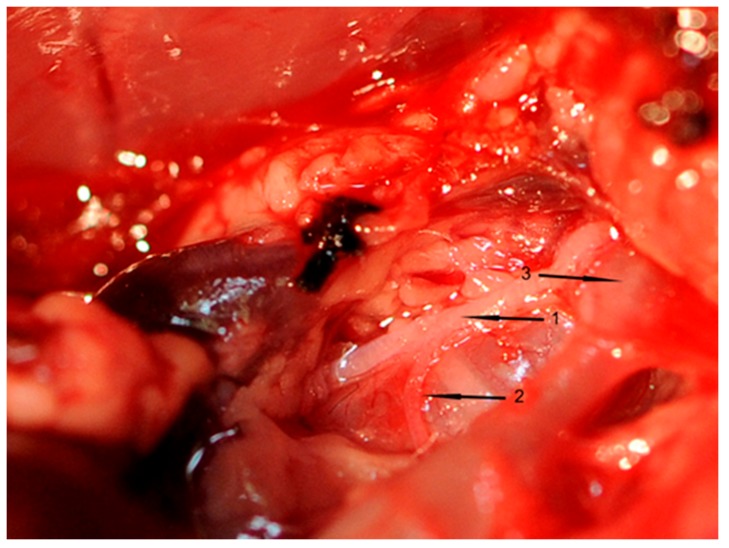
Representative figure of right cardiac vagus nerve preparation. **1** = Nervus vagus dexter, **2** = Cardiac branch of the right vagus nerve, **3** = Truncus brachiocephalicus.

All inserted catheters were fixed (6.0 nylon threads) accurately before the rat’s chest was opened. A 3-cm incision was made to divide the skin and muscle layers. An electrocautery device (Aesculap, Tuttlingen, Germany) was used to avoid or stop bleeding from intercostal arteries. The thorax was opened between the first rib to above the diaphragm using scissors. To prepare, loop, and cut the right cardiac vagus nerve (VGX), a small part of the right thymus was resected. Thereafter, the right cardiac nerve branch of each rat was exposed (Figure 7), which was cut in rats of groups II and IV. This surgical procedure took one hour in total.

A respective sham operation was performed in rats of groups I and III without cutting the nerve. After these manipulations, a 30-min steady state interval was maintained without any additional manipulations. To make a gap between surgical procedure and LPS administration is justified by the idea to give the animal defined time interval to recover from surgical procedures for 30 min.

Thereafter, 1 mL of NaCl or LPS was given via the V. femoralis sinistra. After 4 h of LPS or vehicle injection, the animals were sacrificed under general anesthesia by bleeding out. Blood samples were taken, and cardiac tissue was sampled. The rat’s heart was separated into two sections: one half was immediately transferred into RNAlater (Thermo Fisher Scientific, Waltham, MA, USA) for RNA isolation; the other half was immediately transferred to dry ice. All tissue was stored at −80 °C before performing the biochemical analysis.

### 4.3. Blood Sampling

Blood samples (0.1 mL per sampling point) were taken via the A. femoralis at 5 time points: before the surgical procedure was finished (T1), at the end of the steady state phases (T2), 1.5 h (T3) and 3 h (T4) after LPS administration, and immediately before sacrifice (T5).

Blood taken with a glass capillary (10 µL) was immediately used to analyze blood gas [pH value, pCO_2_, pO_2_, bicarbonate (HCO_3_^−^), base excess (BE), hematocrit (Hk), hemoglobin (Hb), potassium (K^+^), glucose (Glu), and lactate (Lac) concentrations using a blood gas analyzing system (Rapid 500, Siemens Healthcare GmbH, Erlangen, Germany)]. Fifty microliters were separated and subsequently frozen at −80 °C to determine CHE [acetylcholinesterase (ACHE) and butyrylcholinesterase (BuCHE)] activities. The remaining blood was rapidly centrifuged at 7000 rpm at 20 °C for 15 min and the supernatant was separated and frozen at −80 °C to assess the biochemical parameters.

### 4.4. Hemodynamic Measurements

For the 4 h of hemodynamic experiments, we chose the closed-chest approach for left ventricular catheterization because of its less invasive character [35]. To optimally maintain the vital functions and to stabilize the animals over the experimental period, the rats’ body temperatures were kept at about 37 °C, and an adequate amount of fluid (2 mL) was given to all animals.

Cardiac function studies of the left ventricle were performed via a pressure-volume conductance catheter (SPR-838; Millar Instruments, Houston, TX, USA).

As described above, the right carotid artery was exposed and separated from surrounding connective tissue and the vagus nerve. The artery was punctured with a 26-G needle, and the catheter was inserted and fixed with a suture (6.0 nylon thread) and carefully moved into the left ventricle. Pressure-volume signals were digitized with a PowerLab 8/35 signal convertor (ADInstruments, Spechbach, Germany) and recorded continuously using the LabChart7 Pro Software for Windows (ADInstruments, Spechbach, Germany). Hemodynamic parameters (heart frequency [HF], cardiac output [CO], end-diastolic volume [EDV], and mean arterial blood pressure [MAP]) were determined. For each animal, parallel conductance catheter calibration was performed; here, 20 mL of hypertonic saline (10%) was administered. After the experiment, the cuvettes were calibrated with freshly heparinized, warm blood to obtain absolute volume values and the recorded data evaluated with MPVS Ultra (Millar Instruments, Houston, TX, USA).

### 4.5. Lipopolysaccharide (LPS) Administration

Daily LPS that was freshly prepared daily (*E. coli* 0111:B4; InvivoGen, San Diego, CA, USA) or vehicle (0.9% NaCl) was injected intravenously via the femoral vein in the morning between 8 a.m. and 9 a.m. under 3.5 vol. % sevoflurane and oxygen/nitrous oxide (O_2_:N_2_O = 30:70). LPS concentration (1 mg/kg body weight) was adapted according to previous titration experiments and to data from Schulte et al. [17].

### 4.6. Biochemical Analyses

#### 4.6.1. Protein Isolation from Heart Tissue

Frozen heart tissue (right heart ventricle 50–100 mg) was homogenized with an ultrasonic homogenizer (Bandelin Sonopuls; Berlin, Germany) in 500 µL potassium phosphate buffer (50 mM, pH 7.4) with protease inhibitor (Roche, Mannheim, Germany) for 30 s. After centrifugation, the supernatant was collected and total protein concentration was determined using the Expedeon BradfordRed Kit (Expedeon Inc., San Diego, CA, USA) with GE Health Ultrospec 7000 Spectrophotometer (Fisher Scientific, Loughborough, UK).

#### 4.6.2. Cytokine Enzyme-Linked Immunosorbent Assay (ELISA)

The concentration of proinflammatory cytokines was determined in plasma samples and in the right ventricle using the following enzyme-linked immunosorbent assay (ELISA) test kits: Quantikine Rat TNF-α (RTA00), interleukin 6 (IL-6; R6000B), and interleukin 1β (IL-1ß; RLB00) (R&D Systems, Wiesbaden, Germany). We performed the analyses according to the manufacturer’s instructions. For tissue samples, cytokine levels were related to total protein content.

RNA isolation, complementary DNA synthesis, and quantitative real-time polymerase chain reaction (RT-PCR).

For RNA extraction, the left ventricle (50–100 mg) was homogenized with a bead mill (Precellys Evolution; Bertin Corp., Rockville, MD, USA) in 1 mL TRIzol^®^ (Ambion, Carlsbad, CA, USA) and then stored at room temperature for 5 min. After the incubation process, the supernatant was transferred to a new Eppendorf tube and 0.2 mL chloroform (Roth, Karlsruhe, Germany) was added to the homogenate, which was mixed and allowed to stand at room temperature for another 3 min. After incubation, the sample was centrifuged again at 12,000× *g* for 15 min at 4 °C. The aqueous phase was carefully removed and transferred to a new tube. An equal volume of 70% ethanol was added. Afterward, the sample was loaded on an RNeasy Plus Mini Kit column (Qiagen, Hilden, Germany). At this step, isolation was performed according to the manufacturer’s protocol, and RNA was stored at −80 °C. For analysis, 1 mg of RNA was reverse transcribed using the QuantiTect Reverse Transcription Kit (Qiagen).

Quantitative real-time polymerase chain reaction (qRT-PCR) was performed using TaqMan Gene Expression Master Mix (Applied Biosystems, Foster City, CA, USA). Assay numbers were as follows: Rn00580432_m1 (IL-1β), Rn01410330_m1 (IL-6) and Rn01525859_g1 (tumor necrosis factor alpha [TNF-α]), and Rn1775763_g1 (glyceraldehyde-3-phosphate dehydrogenase) (Trizol Reagent, Sigma-Aldrich, Munich, Germany). For data analysis, the Delta CT (cycles of thresholds) method was used (2^−[CT *Gene of interest* − CT *GAPDH*]).

### 4.7. Determination of Acetylcholine Concentration and Cholinesterase Activity 

Blood ACHE and BuCHE were measured spectrophotometrically by applying the method of Ellman et al. [36] with some modifications. Here, 1 µL of blood was mixed with 40 µL of 0.001% heparin and 0.1% H_2_O_2_ for hemolysis and subsequently mixed with 725 µL of a solution containing 200 mM potassium phosphate buffer, pH 7.0, 50 mM NaCl, and 1 mM EDTA. To that we added 25 µL of a solution containing 10 mM DTNB (5,5′-Dithiobis(2-nitrobenzoic acid), 100 mM potassium phosphate buffer, and 18 mM sodium hydrogen carbonate. After 3 min of preincubation at room temperature, 6.25 µL of a substrate solution were added (86 mM acetylthiocholine chloride to measure ACHE activity or 10 mM butyrylthiocholine chloride for BuCHE activity). Formation of free thiocholine and the resulting release of 5-thio-2-nitrobenzoate from DTNB were assayed at 405 nm for 4 min. Specific enzyme activity was expressed as U/g Hb for ACHE and U/L for BuCHE.

To determine the acetylcholine (ACH) and acetylcholine esterase (ACHE) concentrations in the heart, tissue samples of the right ventricle were homogenized in 0.5 mL of 50 mM sodium phosphate buffer (pH 7.7) including a protease inhibitor cocktail in an ultrasonic homogenizer. Thereafter, the homogenates were centrifuged at 13,000 rpm at 4 °C for 5 min. ACH concentration was measured in the supernatant after dilution with a specific reaction buffer to prevent ACH hydrolysis using the Amplex^®^ Red Acetylcholine Assay kit (A12217, Thermo Fisher Scientific, Waltham, MA, USA). Protein was measured according to Lowry et al. [37]. Serum bovine albumin was used as standard.

### 4.8. Determination of Plasma Corticosterone

Corticosterone concentration was assessed in homogenates of the hippocampus by radioimmunoassay (RIA, Arbor Assay DetectX, Ann Arbor, MI, USA) after extraction with diethyl ether and ethanol as described in the manufacturer’s instructions. Intra-assay and interassay variations were under 10%.

### 4.9. Statistical Analysis

All measurements were performed by an independent investigator blinded to the experimental conditions. If the data were distributed normally, we presented data as mean; if the data were distributed not normally after Kolmogorov Smirnoff analysis, the data were presented as median. Thus, results are presented as mean with standard deviation (SD) in the tables and in the text. Data in the figures are presented as median (IQR = Inter Quartile Range) or as mean values ± SD.

Differences within or between normally distributed data were subjected to an analysis of variance (ANOVA) using SPSS v.22.0 (SPSS IBM, Chicago, IL, USA) followed by post-hoc Fisher’s LSD test. Two time points were compared using Student’s *t*-test.

Nonparametrically distributed data were analyzed with Kruskel-Wallis test followed by Mann-Whitney *U* test. Results were considered significant at *p* ≤ 0.05.

## 5. Conclusions

The beneficial effect of cholinergic stimulation during inflammation is indisputable and has been shown in a variety of conditions. Assuming that there is no splenic involvement in this “reflex” and reconsidering the early experiments of Otto Loewi, we put the heart in the center of interest. Contrary to the assumption that ventricular myocardium is only sparsely innervated by the N. vagus, we showed significant changes in heart ACH and ACHE after ablation of the right cardiac vagus nerve. However, we did not demonstrate a significant role of the heart in the CAP.

## Figures and Tables

**Table 1:** Intraoperative blood gas analysis at the end of the experiments.

	pH	pCO_2_ (mm Hg)	pO_2_ (mm Hg)	HCO_3_^−^ (mmol/L)	BE (mmol/L)	Hk (%)	Hb (g/dL)	K^+^ (mmol/L)	Glucose (mg/dL)	Lactate (mmol/L)
(I) Sham saline (*n =* 7)	7.30 (0.03)	48.59 (8.68)	79.66 (9.65)	23.11 (2.53)	−3.53 (1.67)	36.14 (1.77)	12.31 (0.63)	5.93 (0.62)	135.43 (19.32)	1.09 (0.09)
(II) VGX saline (*n =* 7)	7.30 (0.07)	46.94 (9.15)	88.70 (10.82)	22.31 (1.70)	−4.14 (1.47)	38.14 (2.27)	12.94 (0.82)	5.95 (0.77)	145.57 (29.75)	1.13 (0.32)
(III) Sham LPS (*n =* 9)	7.06 (0.16) ^§^	51.89 (16.12)	71.81 (28.93)	14.62 (4.89) ^§^	−15.44 (6.75) ^§^	34.67 (3.50)	11.76 (1.18)	8.52 (2.32) ^§^	57.67 (10.26) ^§^	7.19 (4.83) ^§^
(IV) VGX LPS (*n =* 9)	6.99 (0.14) ^§^	45.38 (10.79)	100.16 (15.16)	10.96 (3.49) ^§^	−20.01 (5.57) ^§^	36.13 (3.36)	12.30 (1.10)	9.59 (1.54) ^§^	23.00 (0.10) ^§^	9.20 (4.05) ^§^
*p*-value ^§^*: III* vs. *I and/or IV* vs. *II*	*LPS* < *0.05*	*n.s.*	*n.s.*	*LPS* < *0.05*	*LPS* < *0.05*	*n.s.*	*n.s.*	*LPS* < *0.05*	*LPS* < *0.05*	*LPS* < *0.05*

Data from blood gas analysis are presented as mean (SD) at the end of the experiments (time point T5). Selective right cardiac vagus resection (VGX) did not show a significant effect (II versus I and IV versus III, *p* > 0.05, not significant, n.s.). A significant effect was obtained after lipopolysaccharide (LPS) administration compared to rats without LPS (^§^ *p* < 0.05, III versus I and IV versus II). Abbreviation: BE: base excess, HK: hematocrit, Hb: hemoglobin, vs.: versus.

**Table 2:** Hemodynamic parameters.

Mean (SD)		T1	T5
MAP (mmHg)	Sham saline	70.7 *(7.8)*	65.2 *(6.5)*
	(II) VGX saline	71.5 *(6.3)*	64.7 *(9.8)*
	(III) Sham LPS	68.2 *(9.5)*	41.5 *(10.3)* ^§^
	(IV) VGX LPS	70.8 *(6.6)*	37.7 *(5.9)* ^§^
	*p*_1_^§^*: III* vs. *I and/or IV* vs. *II*	*n.s.*	^§^ < *0.001,* ^§^ < *0.001*
HF (1/min)	(I) Sham saline	339.8 *(22.2)*	364.3 *(9.0)*
	(II) VGX saline	333.2 *(18.5)*	339.4 *(21.8)* ^#^
	(III) Sham LPS	328.6 *(17.4)*	380.6 *(32.1)*
	(IV) VGX LPS	342.0 *(16.2)*	323.2 *(60.1)* ^##^
	*p*_2_^#^*: II* vs. *I and/or* ^##^ *IV* vs. *III*	*n.s.*	^#^ *= 0.017,* ^##^ *= 0.040*
CO (µL/min)	Sham saline	26565.7 *(6588.9)*	42748.5 *(3488.0)*
	(II) VGX saline	23037.1 *(8397.5)*	31891.4 *(10651.0)* ^#^
	(III) Sham LPS	26585.5 *(7948.9)*	40450.0 *(21760.8)*
	(IV) VGX LPS	21030.3 *(11060.5)*	29831.2 *(11868.7)*
	*p*_2_^#^*: II* vs. *I*	*n.s.*	^#^ *= 0.038*
EDV (µL)	Sham saline	92.0 *(37.4)*	125.5 *(45.5)*
	(II) VGX saline	116.2 *(57.8)*	177.8 *(35.0)* ^#^
	(III) Sham LPS	143.1 *(43.8)*	123.8 *(49.1)*
	(IV) VGX LPS	82.5 *(74.9)*	106.7 *(79.8)* ^§^
	*p*_1_^§^*: IV* vs. *II p*_2_^#^*: II* vs. *I*	*n.s.*	^#^ *= 0.05,* ^§^ *= 0.029*

Data (MAP: mean arterial blood pressure, HF: heart frequency, CO: cardiac output, EDV: enddiastolic volume) measured via Millar catheter are presented as means (SD) starting before LPS injection (T1) until the end of experiments (4 h after LPS injection, T5). No significant differences (*p* > 0.05, n.s.) between the treatment groups were observed at baseline (T1). *p*_1_: significant treatment effect ^§^: LPS versus saline; *p*_2_*:* significant treatment effect ^#^ and ^##^: VGX versus sham. *n.s*.: non-significant.
